# Gender-Disparities in Adults with Type 1 Diabetes: More Than a Quality of Care Issue. A Cross-Sectional Observational Study from the AMD Annals Initiative

**DOI:** 10.1371/journal.pone.0162960

**Published:** 2016-10-03

**Authors:** Valeria Manicardi, Giuseppina Russo, Angela Napoli, Elisabetta Torlone, Patrizia Li Volsi, Carlo Bruno Giorda, Nicoletta Musacchio, Antonio Nicolucci, Concetta Suraci, Giuseppe Lucisano, Maria Chiara Rossi, AMD Annals Study Group

**Affiliations:** 1 Department of Internal Medicine, Montecchio Hospital Local Health Authority of Reggio Emilia, Reggio Emilia, Italy; 2 Department of clinical and experimental medicine, University of Messina, Messina, Italy; 3 Department of Clinical and Molecular Medicine, Faculty of Medicine and Psychology, S. Andrea Hospital, Sapienza University, Rome, Italy; 4 Department of Internal Medicine, Endocrinology and Metabolism, S. Maria della Misericordia Hospital, Perugia, Italy; 5 S.S.D. Diabetes Unit, AAS5, Pordenone, Italy; 6 Diabetes and Metabolism Unit, ASL TO5, Chieri, Italy; 7 UOS Integrating Primary and Specialist Care, A.O. ICP, Milano, Italy; 8 CORESEARCH SRL—Center for Outcomes Research and clinical Epidemiology, Pescara, Italy; 9 Diabetes and Metabolism Unit, Sandro Pertini Hospital, Rome, Italy; University of Chieti, ITALY

## Abstract

We evaluated gender-differences in quality of type 1 diabetes (T1DM) care. Starting from electronic medical records of 300 centers, 5 process indicators, 3 favorable and 6 unfavorable intermediate outcomes, 6 treatment intensity/appropriateness measures and an overall quality score were measured. The likelihood of women vs. men (reference class) to be monitored, to reach outcomes, or to be treated has been investigated through multilevel logistic regression analyses; results are expressed as Odd Ratios (ORs) and 95% confidence intervals (95%CIs). The inter-center variability in the achievement of the unfavorable outcomes was also investigated. Overall, 28,802 subjects were analyzed (45.5% women). Women and men had similar age (44.5±16.0 vs. 45.0±17.0 years) and diabetes duration (18.3±13.0 vs. 18.8±13.0 years). No between-gender differences were found in process indicators. As for intermediate outcomes, women showed 33% higher likelihood of having HbA1c ≥8.0% (OR = 1.33; 95%CI: 1.25–1.43), 29% lower risk of blood pressure ≥140/90 mmHg (OR = 0.71; 95%CI: 0.65–0.77) and 27% lower risk of micro/macroalbuminuria (OR = 0.73; 95%CI: 0.65–0.81) than men, while BMI, LDL-c and GFR did not significantly differ; treatment intensity/appropriateness was not systematically different between genders; overall quality score was similar in men and women. Consistently across centers a larger proportion of women than men had HbA1c ≥8.0%, while a smaller proportion had BP ≥140/90 mmHg. No gender-disparities were found in process measures and improvements are required in both genders. The systematic worse metabolic control in women and worse blood pressure in men suggest that pathophysiologic differences rather than the care provided might explain these differences.

## Introduction

Diabetes reverses the sex-related relationships for cardiovascular morbidity and mortality observed in the non-diabetic population and it is particularly harmful in women. Thus, cardiovascular (CV) disease relative risk associated with diabetes is higher in both women with type 1 (T1DM) and type 2 (T2DM) diabetes than in men [1,2]. In particular, women with T1DM have a roughly 40% greater excess risk of all-cause mortality, and twice the excess risk of fatal and nonfatal vascular events, compared to men with type 1 diabetes [3].

The pathophysiologic mechanisms underlying this excess risk are still partly unknown; certainly, differences in accessibility and quality of care can contribute to these disparities [4,5].

The current availability of big databases in several countries can allow a more accurate description of quality of care in the two genders and inspire pre-clinical and clinical research [6–8]. In Italy, gender-medicine has become an integral part of a wider monitoring and continuous improvement initiative in place since 2006 [9–12]. The AMD Annals initiative, which involves approximately one-third of all the diabetes outpatient clinics operating within the national healthcare system, allows the monitoring of a large set of process (i.e. diagnostic procedures and pharmacological prescriptions) and outcome indicators (i.e. clinical results) and the use of specific classes of drugs in about 500,000 patients with T1DM or T2DM, with the aim of examining strengths and limitations of the current diabetes care. Database is highly representative of the clinical practice since it includes all subjects seen at least once in the participating centers during a year, without applying any exclusion criteria. This activity has led to progressive improvements in the quality of care [13,14].

Within this initiative, we have recently documented that T2DM women have an overall worse CV risk profile (i.e. worse outcome indicators) than men in the context of a comparable care provided (i.e. similar process indicators) [11,12], consistently with other reports [15,16]. Data suggested that there is a gap between what is done and what is obtained in terms of outcomes. If we exclude differences in the care provided, we can thus hypothesize that other factors come into play in influencing the outcomes, namely patho-physiologic and/or socio-cultural factors.

Gender-differences in adults with T1DM have been seldom investigated [1,6–8]. Starting from the AMD Annals database, we adopted a similar approach applied in T2DM [11] to investigate whether gender differences in quality of care (i.e. process and treatment indicators) for type 1 diabetes exist in Italy. We also investigated the role of differences in the care provided vs. other hypothetical factors (e.g. patho-physiologic and/or socio-cultural factors) in determining different outcomes indicators and a different distribution of CV risk factors between men and women.

## Materials and Methods

### The AMD Annals initiative

Since 2006, The Italian Association of Clinical Diabetologists (Associazione Medici Diabetologi—AMD) promoted a continuous quality improvement initiative called AMD Annals. In this context, AMD identified a set of process and intermediate outcome indicators to be used for benchmarking activities [9,10]. Furthermore, the use of glucose-lowering, antihypertensive, and lipid-lowering drugs is evaluated.

Centers share the same software for data extraction from electronic medical records. Data are collected in a standardized format (AMD Data File. Database is anonymous by design. Information is extracted by the medical records system without any data allowing the identification of patients and centers. Both are identified by numeric codes and analysis is centralized and based on aggregated data [9,10]. Given the nature of the study, the Italian regulation did not require an ethics approval. The entire project is conducted without allocation of extra resources or financial incentives, but simply through a physician-led effort, made possible by the commitment of the specialists involved.

### Sample selection

All patients with diagnosis of T1DM were included. Clinical data collected during the year 2011 were extracted from electronic medical records (S1 Fig). In case of multiple records collected during the year for the same patient, the last available value was included in the quality of care profiling [9–13].

### Quality of care indicators

Process measures are expressed as percentages of patients monitored at least once during the previous 12 months for the following parameters: glycated hemoglobin (HbA1c), blood pressure (BP), lipid profile (LDL cholesterol or total and HDL cholesterol and triglycerides), renal function, and eye examination [9–14].

Intermediate outcome measures include the proportion of patients with satisfactory values as well as the percentage of those with unacceptably high values. Outcomes are considered satisfactory if HbA1c levels are ≤7.0% (≤53 mmol/mol), blood pressure values are ≤130/80 mmHg, and LDL cholesterol (LDL-c) levels are <100 mg/dl. Unsatisfactory outcomes include HbA1c levels >8.0%, blood pressure values ≥140/90 mmHg, LDL levels ≥130 mg/dl, BMI ≥30 Kg/m^2^, presence of micro/macroalbuminuria (MAU), and glomerular filtration rate (GFR) ≤60 ml/min. Indicators of treatment intensity/appropriateness are also measured, taking into consideration the use of pharmacologic treatments in relation to the achievement of the targets: no lipid-lowering agents despite LDL-c ≥130 mg/dl, no antihypertensive treatments despite BP≥140/90 mmHg, no angiotensin converting enzyme inhibitors (ACE-Is) and/or angiotensin receptor blockers (ARBs) despite MAU, HbA1c> = 9.0% in spite of insulin therapy, LDL-c ≥130 mg/dl in spite of lipid-lowering treatment, and BP ≥140/90 mmHg in spite of antihypertensive treatment [9–14].

Finally, a quality of care summary score (Q score) was calculated which is based on a combination of process and outcome indicators based on levels and treatment of HbA1c, blood pressure, LDL-cholesterol and MAU [13,14]. The score ranges between 0 and 40; the higher the score, the better the quality of care. Two validation studies [17,18] documented that the risk to develop a new CV event was 80% higher in patients with a score <15 and 20% higher in those with a score between 15 and 25, as compared to those with a score >25.

The denominators for the different quality indicators vary according to the availability of the information in the index year. No missing imputation was performed.

LDL-c was estimated by the Friedwald equation. MAU was defined as albumin excretion rate ≥20 mcg/min, albumin/creatinine ratio >2.5 (men) or >3.5 (women) mg/mmol or urine albumin >30 mg/l. GFR was calculated with the Chronic Kidney Disease Epidemiology Collaboration (CKD-Epi) formula [19].

### Statistical analyses

Patients characteristics and quality indicators according to gender are described as mean and standard deviation or frequencies. Patient characteristics and quality indicators by gender were compared using the Mann–Whitney U-test for continuous variables and the Mantel–Haenszel χ^2^ test for categorical ones.

The likelihood of women as compared to men (reference class) to be monitored for specific clinical parameters, to reach specific clinical outcomes and to be treated with specific classes of drugs has been investigated through multilevel logistic regression analyses, adjusted for age, diabetes duration, BMI, smoking and, in a separate model, also for clustering effect; participating diabetes outpatient clinics accounted for the clusters.

Multilevel analyses were additionally adjusted for process indicators (i.e. monitoring and appropriateness indicators) associated with each outcome. We tested these variables as center-level covariates.

Results of multilevel analyses are expressed as odds ratio and their 95% confidence intervals.

For each indicator, we estimated the intraclass correlation coefficient (ICC) to evaluate the extent to which the indicator varies between centers as compared to within-center variation, taking patient characteristics into account [20]. The higher the ICC, the greater the influence of the center level on the quality of care indicator.

## Results and Discussion

Overall, 28,802 patients with T1DM referred to 300 diabetes outpatient centers during the year 2011 were evaluated: 13,094 (45.5%) women and 15,708 (54.5%) men. Patient characteristics according to gender are summarized in Table 1.

**Table 1:** Patients characteristics according to gender.

Patient characteristics	M	F	p-value
N	15,708	13,094	
%	54.5	45.5	
Age (yrs)	44.5±16.0	45.0±17.0	0.17
BMI (Kg/m^2^)	25.0±3.7	24.2±4.3	<0.0001
Smokers (%)	31.8	22.7	<0.0001
Diabetes duration (yrs)	18.3±13.0	18.8±13.0	0.0004
Diabetes duration classes (%)			
≤2	10.7	9.5	0.0006
2–5	8.9	8.4	
5–10	14.3	13.7	
10–20	26.3	27.2	
>20	39.8	41.2	
HbA1c (%)	8.0±1.5	8.2±1.5	<0.0001
HbA1c (mmol/mol)	63.9±16.4	66.1±16.4	<0.0001
Total cholesterol (mg/dl)	184±36	192±35	<0.0001
HDL cholesterol (mg/dl)	57±15	67±16	<0.0001
LDL cholesterol (mg/dl)	108±30	108±29	0.70
Triglycerides (mg/dl)	97±89	83±56	<0.0001
Systolic blood pressure (mmHg)	128±17	124±18	<0.0001
Diastolic blood pressure (mmHg)	76±9	73±9	<0.0001
Diabetes treatment (%)			
Continuous subcutaneous insulin infusion (CSII)	13.9	19.6	<0.0001
Multiple daily injection (MDI)	86.1	80.4	
Lipid-lowering agents (%)	25.4	23.5	0.0002
Antihypertensive treatment (%)	28.7	26.1	<0.0001

Data show some small differences in between-gender clinical characteristics. Mean age and diabetes duration were similar in the two groups, with 40% of subjects with diabetes duration >20 years. Smoking was more prevalent and BMI was higher in men than in women. The two genders also showed slight differences in glucose control, lipid profile and blood pressure. Women were more often treated with continuous subcutaneous insulin infusion (CSII) than men (19.6% vs. 13.9%), but less frequently treated with lipid-lowering agents and antihypertensive drugs (Table 1).

Quality indicators are shown in Table 2.

**Table 2:** Quality indicators of diabetes care according to gender.

Process indicators	M (%)	F (%)	p-value
HbA1c	93.5	93.7	0.45
Lipid profile	71.5	71.8	0.47
Blood pressure	75.8	76.3	0.34
Renal function	50.8	51.4	0.27
Eye examination	41.0	41.3	0.55
**Favorable outcome indicators**			
HbA1c ≤7.0% (≤53 mmol/mol)	25.6	20.4	<0.0001
LDL-C <100 mg/dl	41.4	41.5	0.91
BP ≤130/80 mmHg	61.5	69.5	<0.0001
**Unfavorable outcome indicators**			
HbA1c >8.0% (>64 mmol/mol)	41.6	47.3	<0.0001
LDL-C ≥130 mg/dl	22.1	20.7	0.02
BP ≥140/90 mmHg	31.5	25.2	<0.0001
BMI ≥30 Kg/m2	8.7	9.8	0.002
GFR ≤60 ml/min	7.8	9.6	<0.0001
MAU	30.1	24.7	<0.0001
**Indicators of treatment intensity/appropriateness**			
No lipid-lowering agents despite LDL-c ≥130 mg/dl	68.2	71.1	0.04
No antihypertensive treatments despite BP ≥140/90 mmHg	49.8	47.4	0.06
No ACE-I and/or ARBs despite MAU	8.7	8.9	0.58
LDL-c ≥130 mg/dl in spite of lipid-lowering treatment	23.1	21.7	0.22
BP ≥140/90 mmHg in spite of antihypertensive treatment	50.8	47.6	0.01
**Overall quality of care**			
Q score <15	7.5	7.6	0.68
Q score >25	40.6	41.5	0.14

Crude percentages show no gender-differences in the process indicators. Men showed better glucose control, women showed better blood pressure control and LDL-c levels; MAU was more frequent in men, while GFR< = 60 ml/min was more prevalent in women. No lipid-treatment despite LDL-c> = 130 mg/dl was more frequent in women, whereas uncontrolled blood pressure in spite of antihypertensive treatment was more frequent in men. Q score was similar in the two genders.

In terms of crude and adjusted likelihood (Table 3), no statistically significant differences emerged in terms of process indicators, while several disparities were found in the proportions of patients reaching favorable and unfavorable outcomes.

**Table 3:** Quality indicators of diabetes care according to gender. The first three columns show the likelihood (odds ratios and their 95% confidence intervals) of women as compared to men (reference class) to be monitored for specific clinical parameters, to reach specific clinical outcomes and to be treated with specific classes of drugs. Odds ratios are crude, adjusted for patient characteristics only (age, diabetes duration, BMI, and smoking), and for patient characteristics and clustering effect. Statistically significant differences are in bold. The fourth column shows intra-class correlation coefficients (ICC). The higher the ICC, the greater the influence of the center level on the quality of care indicator. The fifth column shows the results of the multilevel analyses additionally adjusted for process indicators (i.e. monitoring and appropriateness indicators associated with each outcome).

	Crude likelihood	Adjusted for Age, Duration, BMI and Smoke	Adjusted for Age, Duration, BMI and Smoke, and Clustering effect	Intra-class correlations (ICC)	Adjusted for Age, Duration, BMI and Smoke, Clustering effect + Process/treatment indicators specific for the outcome	Intra-class correlations (ICC)
	OR (95% CI)	OR (95% CI)	OR (95% CI)		OR (95% CI)	
**Favorable outcome indicators**						
HbA1c ≤7.0% (≤53 mmol/mol)	**0.75 (0.70–0.79)**	**0.71 (0.65–0.77)**	**0.70 (0.64–0.76)**	0.04	**0.70 (0.64–0.76)**	0.04
LDL-C <100 mg/dl	**1.00 (0.95–1.06)**	**0.99 (0.92–1.07)**	**0.99 (0.92–1.07)**	0.03	**0.97 (0.90–1.05)**	0.01
BP<130/80 mmHg	**1.42 (1.35–1.51)**	**1.40 (1.29–1.51)**	**1.44 (1.33–1.56)**	0.12	**1.44 (1.33–1.56)**	0.04
**Unfavorable outcome indicators**						
HbA1c >8.0% (>64 mmol/mol)	**1.26 (1.20–1.32)**	**1.32 (1.23–1.41)**	**1.33 (1.25–1.43)**	0.03	**1.33 (1.25–1.43)**	0.03
LDL-C ≥130 mg/dl	0.92 (0.86–0.99)	0.95 (0.87–1.04)	0.96 (0.88–1.05)	0.04	0.97 (0.89–1.07)	0.01
BP≥140/90 mmHg	**0.73 (0.69–0.78)**	**0.72 (0.66–0.78)**	**0.71 (0.65–0.77)**	0.11	**0.71 (0.65–0.77)**	0.02
BMI≥30 Kg/m2	**1.15 (1.05–1.25)**	1.06 (0.95–1.19)	1.07 (0.95–1.19)	0.05	**N/A**	
GFR≤60 ml/min	**1.26 (1.14–1.39)**	1.09 (0.93–1.27)	1.09 (0.93–1.27)	0.01	1.09 (0.93–1.27)	0.01
MAU	**0.76 (0.71–0.82)**	**0.77 (0.70–0.85)**	**0.73 (0.65–0.81)**	0.35	**0.73 (0.65–0.81)**	0.27

In the fully adjusted models, as compared to men, women showed a 30% lower likelihood to reach HbA1c ≤7.0%, a 44% higher likelihood to reach LDL-c <100 mg/dl and a 37% higher likelihood to reach BP ≤130/80 mmHg. These findings are mirrored by those relative to unfavorable outcomes: as compared to men, women showed a 33% higher likelihood of having HbA1c ≥8.0%, 29% lower risk of BP ≥140/90 mmHg and a 27% lower risk of MAU (Table 3). Differences in BMI and GFR were lost after adjustment.

In terms of intensity/appropriateness of treatment, women had a 49% higher likelihood not to be treated with lipid-lowering agents despite high levels of LDL-c and a 17% higher likelihood not to be treated with antihypertensive drugs despite poor levels of BP. When evaluating the likelihood of not reaching the target despite the treatment, in the fully adjusted models women had a 29% higher likelihood to have HbA1c >9.0% and a 18% lower likelihood of having BP levels ≥140/90 mmHg in spite of treatments (Table 3).

In terms of overall quality of care, data show similar proportions of patients with Q score <15 and >25 in the two genders.

The contribution of between center variability was substantial for process measures, as documented by ICC >0.20, and moderate for other indicators, as documented by lower ICC values (Table 3).

In the additional models performed by adjusting each outcome also for its corresponding monitoring indicator and, if available, intensity/appropriateness indicators, ORs were unchanged and ICC reduced (Table 3).

**Fig 1:**
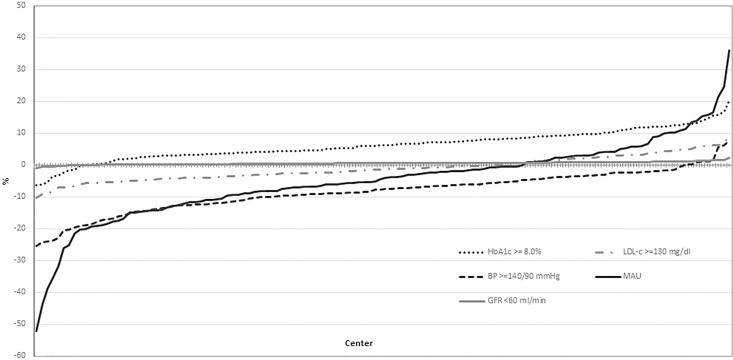
Inter-center variability in the percentage difference of men and women who reach the unfavorable intermediate outcomes. The inter-center variability in the difference between men and women achieving the different unfavorable targets was investigated using multilevel models adjusted for age, diabetes duration, BMI, and clustering effect. For each center, the estimated difference between men and women who reached the outcome indicator was calculated. These differences were ranked from the lowest to the highest and reported in the graph. The dotted line represents the absence of between gender differences. When the value for a center is above the dotted line, the percentage of individuals with unfavourable outcome is higher in women than in men; when the value is below the dotted line, the percentage is lower in women than in men. When most of the values for one specific indicator are above or below the dotted line, this indicates the presence of a between-gender difference for that outcome in the majority of the centers.

Finally, we examined the between center variability in the proportion of men and women reaching the unfavorable intermediate outcomes (Fig 1).

In almost all the centers, the percentage of women with HbA1c ≥8.0% was higher than men, while the proportion of patients with BP ≥140/90 mmHg was lower in women. The proportion of individuals with MAU was also lower in women in the vast majority of centers, with elevated inter-center variability. No relevant difference was found in terms of inter-center variability for LDL-c and no difference for reduced GFR.

Finally, we performed two sensitivity analyses to assess data robustness: we evaluated patient characteristics utilizing the “first value” recorded in the index year for each parameter and utilizing the sub-sample of subjects who had full availability of all data on outcomes indicators and adjustment covariates (i.e. the sample selected by the multivariate and multilevel models, i.e. 5,856 men and 4,768 women). Patient characteristics totally overlap in all the examined samples (data not shown).

### Main findings

We provided a comprehensive evaluation of the quality of T1DM care in Italy, adopting a large set of indicators. Our data show a lower likelihood to reach a good metabolic control for women, and a lower likelihood of achieving blood pressure targets for men. These gender-differences persisted after adjustment for patient socio-demographic and clinical characteristics and clustering effect, and were consistently found in almost all the centers. Since process indicators did not show any gender-difference, these findings can hardly be explained by gender-oriented attitudes of physicians; nevertheless, differential behaviors, for example in physician-patient communication, cannot be totally excluded.

Our data show that T1DM women had a 29% higher risk of having a HbA1c>9% and, although women showed a larger use of CSII vs. MDI, the consistency of bad metabolic control in the female gender across all the centers suggests that causes other than the type of treatment may play a role. On the other hand, the larger use of CSII in women may be the consequence of the effort to improve their metabolic control.

Conversely, women showed a better BP control, although they were less intensively treated with antihypertensive agents; notably, when treated, women reached more frequently the desired outcomes.

Similarly, in the majority of the centers, women were less likely than men to have elevated LDL-C levels despite showing a lower likelihood to be treated with lipid-lowering agents in the presence of elevated values.

Q score, which is an overall quality of care indicator, was similar in the two genders, although different components contributed to the score (i.e. worse metabolic control in women and worse blood pressure in men).

### Comparison with existing knowledge

We previously investigated through asimilar approach gender disparities in the care provided to people with T2DM [9]; data suggested a greater difficulty in reaching glucose and LDL-C targets in women, which was not explained by a different quality of care in terms of monitoring and appropriate prescriptions, that were similar in the two genders. Our current data show that gender differences also exist in T1DM, although with some relevant differences with those reported in T2DM. Thus, while T2DM women showed a worse glucose and lipid control, in women with T1DM glucose control was worse, consistently with T2DM, but BP and lipid profile were better than in men. Notably, in T2DM the Q score was significantly poorer in women [11], while no between gender differences emerged for T1DM in the overall quality of care.

Gender-differences in children and young adults with type 1 diabetes were also described in the German diabetes documentation and quality management system (DPV) [6] involving 27,358 individuals: consistent with our results, HbA1c levels were higher in female gender and hypertension and smoking were more frequent in males. On the other hand, patterns of care in adults with type 1 diabetes have been seldom investigated. Recently, data deriving from two large databases were published: the first study showed that the proportions of patients with HbA1c <58 mmol/mol (<7.5%) varied from 20.5% to 53.6% among 229,677 people aged ≥25 years in 20 regional or national registries, with women showing a worse metabolic control [7]; the other study, involving 15,351 patients from general practices in Scotland, showed that men had lower HbA1c levels and higher BP than women, whereas data on BMI and lipid profile favored men [8].

All these analyses consistently show a worse glucose control in T1DM women and worse BP levels in men, and support the need for further investigation on causes of these gender disparities other than sex-differences in medication use or diagnostic procedures. Furthermore, the larger prevalence of MAU in men, that can at least partially reflect the higher prevalence of smoking and the poorer blood pressure control, and the finding of a larger percentage of subjects with lower GFR values among women is consistent to what reported in T2DM [11,15].

Large databases on type 1 diabetes populations are now available in several countries, e.g. Germany/Austria, USA, Scotland, and Sweden; these populations have characteristics similar to our sample in terms of diabetes duration, age, and/or distribution by gender [7,8, 21–24], but to our knowledge data on gender-differences in quality of care were not considered.

### Implications for clinical practice

The comprehensive evaluation of a large set of different quality indicators suggests that major improvements in the care provided to adults with T1DM is needed in both genders. Almost one in two patients shows HbA1c levels of 8% or more, one in five has LDL-C levels ≥130 mg/dl, and more than one in four shows BP levels ≥140/90 mmHg and MAU. The need for treatment intensification is clearly showed by the high percentages of patients not pharmacologically treated despite elevated LDL-C and blood pressure levels. These findings, together with the detection of substantial between centers variability especially for the prevalence of MAU, will represent the basis for the implementation, also in T1DM, of a continuous quality improvement effort, based on the “best performers” approach [9–10].

Furthermore, although the mean Q score was similar, men and women show different clinical needs. In line with previous studies, we confirm a greater difficulty in reaching an adequate metabolic control and a more frequent adoption of CSII in women than in men. These findings call for a deeper understanding of gender-specific attitudes and barriers and for new gender-based psychosocial approaches. On the other hand, interventions on lifestyle should be intensified primarily in men, especially those aimed at smoking cessation and weight control.

### Implication for research

Previous studies showed a higher relative risk for CVD morbidity and mortality in women than in men with T1DM [3]. Based on our data, this excess risk cannot be explained by differences in medication use or diagnostic procedures and a greater burden of CV risk factors in female vs. male gender. Q score, which is an independent predictor of CV events, was comparable in the two genders. This suggests that a complex interplay among clinical and non-clinical factors may contribute to this excess risk. In this sense, further research on the role of other non-classical CVD risk factors, safety of treatments and/or different genetic, hormonal, cultural, educational, behavioral characteristics in determining gender-differences in short- and long-term outcomes represents a high priority.

### Strengths and limitations

The main strength is the large sample size and the data source, largely representative of the quality of diabetes care provided to people with T1DM in Italy.

Among the limits, we missed information on drugs doses, socio-demographic and socioeconomic characteristics, diabetes macrovascular complications, and patient-centered outcomes which could help understand the reasons for the results obtained. Furthermore, we applied logistic regression. This is the most commonly used method, although some authors suggest that the estimates deriving from logistic regression could be overestimated [25].

## Conclusions

Our data suggest that in T1DM the greater difficulty in reaching good metabolic control in women and good blood pressure control in men can be related to factors other than between gender differences in medication use or diagnostic procedures. On the other hand, physician attitudes can play an important role for all the other indicators. These findings underline the need for a gender-based approach in T1DM care, while further research is required to clarify mechanisms underlying these differences. The regular evaluation of quality of care indicators through initiatives as the AMD Annals is the first fundamental step to identify main areas of interventions and to monitor the desirable improvements during the time.

## Supporting Information

S1 Appendix AMD Annals 2012 Study Group. (DOCX)

S1 Fig Flow-chart of data selection and data availability. (TIF)
