# Supplementary material for: Gender-Disparities in Adults with Type 1 Diabetes: More Than a Quality of Care Issue. A Cross-Sectional Observational Study from the AMD Annals Initiative
Source: PLoS One. 2016 Oct 3;11(10):e0162960. doi: 10.1371/journal.pone.0162960 (PMC5047461; doi:10.1371/journal.pone.0162960)
Supplement: S1 Appendix — (DOCX) [file pone.0162960.s001.docx]

**S1 Appendix: AMD Annals 2012 Study Group**

**Editorial Board (in alphabetical order):** Adolfo Arcangeli - Prato, Antonino Cimino - Brescia, Paolo Di Bartolo - Ravenna, Danila Fava - Roma, Augusto Lovagnini-Scher – Cusano Milanino (MI), Alberto Marangoni – Bassano Del Grappa (VI), Illidio Meloncelli - S. Benedetto del Tronto (AP), Maria Franca Mulas – Oristano, Antonio Nicolucci, Santa Maria Imbaro (CH), Andrea Nogara – Chioggia, Fabio Pellegrini, Santa Maria Imbaro (CH), Maria Chiara Rossi - Santa Maria Imbaro (CH), Salvatore Turco – Napoli, Giacomo Vespasiani - S. Benedetto del Tronto (AP)

**Statistical analysis:** Antonio Nicolucci, Maria Chiara Rossi, Fabio Pellegrini, Giuseppe Lucisano, Basilio Pintaudi, Marco Scardapane, Riccarda Memmo, Elena Pellicciotta, Fondazione Mario Negri Sud, Santa Maria Imbaro (CH).

**Regional Tutors (in alphabetical order by region):** La Penna G, Paciotti V,– Abruzzo; Venezia A – Basilicata; Armentano G, Giovannini C – Calabria; Armentano V, Laudato M, Turco S – Campania; Laffi G, Monesi M - Emilia Romagna; Felace G, Tortul C - Friuli Venezia Giulia; Santantonio G, Suraci C – Lazio; Ghisoni G, Raffa M – Liguria; Genovese S, Lovagnini-Scher CA, Mangone I, Richini D, Rocca A – Lombardia; Garrapa G, Tortato E – Marche; Vitale C – Molise; Comoglio M, Fornengo R – Piemonte; De Cosmo S, Gentile FM - Puglia; Gigante A, Mastinu F – Sardegna; Di Benedetto A, Saitta G – Sicilia; Calabrese M, Orsini P – Toscana; De Blasi G, Romanelli T - Trentino Alto Adige; Cicioni G, Pocciati S – Umbria; Marangoni A, Nogara A – Veneto.

**Participating centres (in alphabetical order by town):** Marilena Lanero, Carla Bergonzini, Maria Grazia Bertero, Alba Rapetti, Gabriella Martini, Monica Tornaghi - Acqui Terme (AL); Antonino Pipitone, Lucio Bellinetti, Gemma Frigato, Daniela Santipolo - Adria (RO); Massimo Boaretto, Luisa Parmesan, Barbara Conte, Iva Manfroi, Fanny Soccol, Nadia Cumina - Agordo (BL); Annamaria Nuzzi, Elisabetta Benedusi Pagliano, Emanuele Fraticelli, Paola Viberti, Antonietta Pasquero - Alba (CN); Adalberto Pagano, Enrico Papini, Marco Chianelli, Filomena Graziano, Irene Misischi, Lucilla Petrucci, Roberta Rinaldi, Stefania Silvagni - Albano Laziale (RM); Maura Rosco - Alberobello (BA); Egle Ansaldi, Francesco Malvicino, Maurizia Battezzati, Paolo Maresca, Marcella Balbo, Clara Palenzona - Alessandria; Giovanni Cicioni, Stefania Venturi - Amelia (TR); Valentino Cherubini, Antonio Iannilli, Martina Biagioni, Chiara Giorgetti, Maria Cristina Alessandrelli, Alessandra Cesaretti, Anna Maria Paparusso - Ancona; Massimo Boemi, Rosa Anna Rabini, Gabriele Brandoni, Federica D'Angelo - Ancona; Maria Luigia De Cicco, Federica Giampetruzzi, Filomena Loconte, Simona Maldera, Stefania Annese, Palma Di Corato, Daniela Strippoli, Tiziana Rizzi - Andria (BAT); Giulio Doveri, Emma Lillaz, Antonio Ciccarelli, Salvatore Giardina, Roberta Courthoud, Paola Mararia, Elisa Pascale, Silvana Flou - Aosta; Grazia Pia Ricciardi, Paola Di Masa - Aprilia (LT); Guido Vitalone, Donatella Setti, Giuseppe Piticchio, Patrizia Contrini, Camilla Mazzola - Arco (TN); Paola Ponzani, Valeria Ghigliotti, Andrea Corsi - Arenzano (GE); Vincenzo Mazzini, Marina Orlandi, Maria Cristina Pollace - Argenta (FE); Stefano Fazion, Maria Luisa Spina, Cinzia Francesconi - Asola (MN); Vincenzo Trosini, Roberto Berardinucci, Katia Dolcetti, Paola Colleluori, Emanuela Cannarsa, Valeria Montanari, Paolo Di Berardino - Atri (TE); Mario Velussi - Aurisina (TS); Vincenzo Paciotti, Pasquale Alfidi, Bruno Verdecchia, Luigina Baliva, Alessia Di Pietro, Giovanna Franchi, Patrizia Luce - Avezzano (AQ); Simona Flora Maldera, Mariangela Manicone, Daniela Strippoli, Ida Console, Rossella Caringella, Anna Leonardini, Michele Giardino, Giuseppe Stornelli, Cristina Palmiotto,Gianluca Besozzi, Daniela Di Marzo, Adriana Lategola,Alessandra Marcotrigiano, Paolo Sciascia - Bari; Alberto Marangoni, Alessandro Pianta, Maria Ferrari, Sara Balzano, Gianpietro Beltranello - Bassano Del Grappa (VI); Massimo Boaretto, Concetta Nadia Aricò, Corradina Alagona, Laura Cervo, Silvia Rossa, Rosella Zanon - Belluno; Franco Travaglino, Rita Graziella Guarnieri - Biella; Maura Rosco, Maria Concetta Di Pace - Bisceglie (BAT); Gilberto Laffi, Silvio Giangiulio, Michele Grimaldi, Giovanna Santacroce, Fernanda Cerrelli, Rita Manini, Adolfo Ciavarella - Bologna; Stefano Zucchini, Claudia Balsamo, Mirella Scipione, Giulio Maltoni, Alessandra Rollo - Bologna; Bruno Fattor, Tiziano Monauni, Florian Amor, Dalia Crazzolara, Gerhard Orion, Michela Cristini, Sylvia Lintner,Johanna Eisath - Bolzano; Laura Chiappini, Sandra Alberti - Bondeno (FE); Stefano Garavelli, Teresa Calari, Paola Marini - Borgo Valsugana (TN); Marco Buschini, Daniela Bonfiglioli, Damiano Mones - Borgomanero (NO); Aldo Giuseppe Morea, Lucia Bondesan, Sandro Perbellini - Bovolone (VR); Emanuele Fraticelli, Mauro Ravera - Bra (CN); Antonino Cimino, Umberto Valentini, Angela Girelli, Liliana Rocca, Barbara Agosti, Emanuela Zarra - Brescia; Gianfranco De Blasi, Michael Bergmann, Franz Plaickner, Alessandra Ferro Ingaglio, Irmgard Pradi, Marianne Piok, Rosmarie Unterkircher, Marion Pichler - Bressanone (BZ); Giuseppina Palamà, Patrizia Palma, Claudia Pisanello, Antonio Trinchera - Brindisi; Brigitte Viehweider - Brunico (BZ); Mauro Giovanni Schiesaro, Giovanni Vita, Beatrice Cenci, Laura Pizzamiglio - Bussolengo (VR); Francesca Spanu, Sandro Cocco, Rasangela Maria Pilosu, Pier Paolo Contini - Cagliari; Giampiero Piras, Roberto Seguro, Marco Songini - Cagliari; Marco Stabilini, Fabrizio Lombardini, Salvatorina Mura, Paola Sulis, Aldo Caddori - Cagliari; Maria Giulia Cartechini, Natalia Busciantella Ricci, Giacoma Agostinelli - Camerino (MC); Antimo Aiello, Maria Rosaria Cristofaro, Lorenzo Cocco, Simonetta Di Vincenzo, Fiorentina D'Amico, Vittoria Di Iorio, Franca Di Ponte, Graziella Giancola, Emanuela Mazzola, Vittoria Niro, Lina Notartomaso, Carmela Sabetta - Campobasso; Annamaria Nuzzi, Claudia Ballauri - Canale (CN); Sergio Casati, Daniela Tettamanzi, Margherita Casartelli, Cosimo Prete, Emanuela Gatti, Luisa Marzorati, Tiziana Cairoli - Cantù (CO); Annelisa Lesina, Francesco Romeo, Carlo Bruno Giorda, - Carmagnola (TO); Anna Vittoria Ciardullo, Daniela Piani, Angela Deroma, Ewa Grazyna Maciejewska, Veronica Baccini - Carpi (MO); Elisabetta Straface - Casalbordino (CH); Giuseppe Pozzuoli, Mario Laudato, Maria Barone - Caserta; Sergio Tondini, Flavia Borgoni - Castel del Piano (GR); Juliette Grosso, Loredana Rossi, Carla Scarsellato, Antonietta Sciulli, Federica De Marco - Castel di Sangro (AQ); Stefano Fazion, Maria Luisa Spina, Cinzia Francesconi - Castel Goffredo (MN); Loris Confortin, Narciso Marin, Mario Lamonica, Elisa Rossi, Anna Pisacane, Vanessa Cavasin - Castelfranco Veneto (TV); Stefano Genovese, Fabiana Locatelli, Valeria Valdes, Flavia Moratti, Valentina Fuso - Castellanza (VA); Giordano Filippi, Monica Camporesi, Mirca Gherardi - Castelnovo Ne' Monti (RE); Sergio Gallana, Gloria Buganza, Adriana Valentini, Ilaria Cera - Castiglione delle Stiviere (MN); Salvatore Gialdino, Rossella Franchino - Castrovillari (CS); Vito Borzì, Concetta Gatta, Riccardo Rapisardi, Salvatore Strano, Maria Calabrò - Catania; Luigi Puccio, Raffaella Vero - Catanzaro; Giuseppe Cheluci, Susy Martucci, Anna Simoncelli, Maria Pia Giammoema, Bruna Seber, Mauro Lozzer, Sonia Andolfatto - Cavalese (TN); Gemma Frigato - Cavarzere (VE); Vincenzo Starnone, Andrea Del Buono, Anna Maria Terracciano, Egidio Gamba - Cellole (CE); Vincenzo Maria Monda, Paola Ramponi, Sandra Alberti, Elisabetta Maccaferri, Flavia Busi - Cento (FE); Francesco Castro, Antonello Guaglianone, Concezione Maccari - Cetraro (CS); Laura Corsi, Giorgio Versari, Maria Rosaria Falivene, Nicoletta Boletto, Simona Corsi, Marisa Delucchi, Simonetta Gasperi, Bruna Ferrari - Chiavari (GE); Carla Origlia, Lisa Marafetti, Carlo Bruno Giorda - Chieri (TO); Ester Vitacolonna, Livia Lina Cavuto, Federica Fraticelli, Matteo D'Angelo - Chieti; Loredana di Nisio, Filomena Simonetti - Chieti; Andrea Nogara, Gianni Ballarin, Angelo Boscolo Bariga, Silvia Di Benedetto - Chioggia (VE); Anna Maria Chiambretti, Riccardo Fornengo, Lidia Di Vito, Maria Divina Pascuzzo, Paola Urli, Monica Albertone, Primo Bassani, Carla Bellaluna, Maria Bonacci, Iolanda Bosso, Daniela Cortale, Vanda De Michelis, Margherita Di Gennaro, Vilma Magliano, Silvia Nieddu, Antonia Stranisci - Chivasso (TO); Alberto Rocca, Paola Galli, Annunziata De Blasi, Monica Castellan, Cristina Cavenaghi, Maria Iagulli - Cinisello Balsamo (MI); Virginia Trinelli, Francesco Caraffa, Daniela Gallo - Ciriè (TO); Roberto Norgiolini, Roberta Celleno, Corrado Campanelli, Daniela Biccheri, Cinzia Bondi, Giuseppa Dadi - Città di Castello (PG); Graziano Santantonio, Luciano Massa, Alessandra Zappaterreno, Lina Lottatori, Elisa Costanzo, Nicoletta Pistola - Civitavecchia (RM); Paolo Ziller, Federica Portolan, Giuseppe Pasolini - Cles (TN); Valerio Di Chiara, Maria Luisa Grata, Elisabetta Bergami, Soccorsa Ciannillo, Navia Curzola - Codigoro (FE); Luigi Capretti, Guglielmina Speroni, Luciano Fugazza - Codogno (LO); Cinzia Massafra - Cologno Monzese (MI); Maria Cristina Cimicchi, Carlo Percudani, Tiziana Risolo, Paola Saccò, Manuela Mori - Colorno (PR); Valerio Di Chiara, Maria Luisa Grata, Elisabetta Bergami, Soccorsa Ciannillo, Navia Curzola - Comacchio (FE); Gian Luigi Gidoni Guarnieri, Diana Piccolo, Franca Giacon, Mariolina Scarpel, Clementina Bravin, Elena De Noni, Vanda Daniotti - Conegliano (TV); Giuseppe Panebianco, Federica Tadiotto, Virgilio Da Tos, Michele D'Ambrosio, Monica Faoncella, Marj Zogno, Cristina Lunardi, Patrizia Crivellaro, Loretta Trivellato - Conselve (PD); Dario Pellizzola, Maria Antonella Zampini, Emanuela Frezzati, Maria Elvira Raminelli, Elena Mari - Copparo (FE); Dario Gaiti, Giuseppina Chierici, Silvia Pilla, Bruna Milli, Monica Camporesi, Paola Caretta, Valeria Vezzani, Melita Copelli - Correggio (RE); Alfonso Longobucco - Cosenza; Patrizia Ruggeri, Sergio Di Lembo, Elisa Carrai, Amalia Degli Innocenti, Lucia Manini, Romano Persico, Cristiana Rossi - Cremona; Enrico Gabellieri, Daniela Sansone, Maria Stella Sbriglia, Giuseppina Comba, Giovanni Valesano, Stefania Bertello, Luisella Ballatore, Salvatore Oleandri - Cuneo; Giampaolo Magro, Francesco Tassone, Donatella Gaviglio - Cuneo; Giuseppe Marelli, Veronica Vilei, Paolo Rumi, Laura Bellato, Mara Fedeli, Antonella Merlini, Giuseppina Pinelli - Desio (MB); Giuseppe Marin, Maria Luisa Contin, Angela De Cata, Anna Patalano, Paola Parlato, Jessica Jacovacci - Dolo (VE); Giuseppe Placentino, Vittoria Zizzari, Sofia Pellanda, Luisa Folchi, Anna Lorenzini - Domodossola (VB); Donata Richini, Stefano Molinari, Chiara Inversini, Roberto Strazzieri, Paolo Stofler, Marco Andreoli, Simona Fornaro - Esine (BS); Giuseppe Panebianco, Federica Tadiotto, Virgilio Da Tos, Michele D'Ambrosio, Monica Faoncella, Marj Zogno, Cristina Lunardi, Patrizia Crivellaro, Loretta Trivellato - Este (PD); Maria Simona Termine, Paolo Di Bartolo - Faenza (RA); Luisella Cotti, Gabriella Garrapa - Fano (PU); Ferruccio D'Incau, Patrizia Lagomanzini, Paola Conte, Fiorina Todesco - Feltre (BL); Paolo Foglini, Elena Tortato, Paola Pantanetti, Sandra Di Marco, Rossana Maricotti, Claudio Bedetta - Fermo; Franco Tomasi, Marcello Monesi, Roberto Graziani, Lucia Penna, Fausto Beretta, Chiara Zamboni - Ferrara; Antonella Guberti, Davide Dazzi, Nicoletta Orlandi - Fidenza (PR); Luca Lione, Gigi Bocchio, Edmondo Bosco, Gianmario Massazza, - Finale Ligure, Loano, Pietra Ligure (SV); Sergio Pocciati - Foligno (PG); Elisa Forte, Concettina Marrocco, Antonella Griffo, Roberta Moschetta - Fondi (LT); Tuccinardi Franco, Francesco De Meo, Elisa Forte, Antonietta Coppola, Raffaele Vallefuoco, Pina Pirolozzi, Vincenzo Placitelli - Gaeta (LT); Arturo Mastropasqua, Paolo Marenco - Garbagnate Milanese (MI); Claudio Taboga, Barbara Catone - Gemona del Friuli (UD); Alberto Aglialoro, Giovanni Careddu, Roberta Guido, Maurizio Patrone, Madina Dagnino, Adelina Massara, Margherita Zecchini, Andrea Corsi - Genova; Micaela Battistini, Francesca Fabbri, Vilma Campora, Patrizia Carosla, Silvana Chinigò, Andrea Corsi - Genova; Guglielmo Ghisoni, Francesca Fabbri, Marina Torresani, Roberto Crovetto, Andrea Corsi - Genova; Giorgio Luciano Viviani, Arianna Durante, Francesca Pais, Vittorio Lilliu - Genova; Maura Rosco, Cornelia Mzzaraco - Gioia del Colle (BA); Ercole D'Ugo, Mariarosaria Squadrone, Tommaso Amenduni, Maria M Iovannisci, Flora Potente, Teresa Delle Donne, Concetta Massa, Luigi Della Penna - Gissi (CH); Silvestro De Berardinis, Ilde Guarnieri, Marina Splendiani, Rosanna Di Giuseppe, Susanna Lupidii, Rosanna Ruggieri, Sabrina D'Antonio, Antonella Valorosi - Giulianova (TE); Barbara Brunato, Roberta Assaloni, Rosalia Loro, Sandro Bucciol, Raimonda Muraro, Roberto Da Ros, Carla Tortul - Gorizia; Maura Rosco, Chiara Lavacca - Gravina (BA); Fabrizio Quadri, Gigliola Sabbatini, Laura Sambuco, Clorinda Santacroce - Grosseto; Giuseppina Chierici, Silvia Pilla, Bruna Milli, Melita Copelli, Lorella Bertelli, Pietro Zanichelli, Simona Bodecchi, Susanna Rovesti - Guastalla (RE); Cecilia Marino, Augusta Micheletti, Annarita Petrelli; Valentina Pasquini - Gubbio (PG); Angelo Corda, Luisa Pisano, Giacomo Guaita, Cinzia Deias, Paola Marino, Giuseppa Cuccu, Tina Poggi, Arianna Marceddu, Susanna Diana - Iglesias (CI); Giuliana Baldassarri, Anna Vacirca - Imola; Maria Antonietta Fois, Valentina Maria Cambuli, Ada Pilia, Rosa Pilia, Giuliana Cao - Isili (CA); Mauro Giovanni Schiesaro, Giovanni Vita, Daniele Bonato, Katia Poletto - Isola della Scala (VR); Stefania Deiana, Francesca Scanu - Ittiri (SS); Giorgio Trevisan - Jesolo (VE); Rossella Iannarelli, Enrico Storelli, Alessandra Sperandio,Filomena Sciarretta, Romana Cialfi - L'Aquila; Alberto Zappa, Stefano Carro, Laura Dreini, Sonia Valesi, Giovanna Cinguetti, Roberta Parentini, Alessandra Guazzetti, Cristina Pennucci - La Spezia; Ercole D'Ugo, Daniela Antenucci, Anita Minnucci, Angela Bosco, Giovanna Angelicola, Angela Di Federico, Giuseppina Di Marco, Rosanna Fresco, Claudia Di Florio - Lanciano (CH); Diletta Ugolotti, Clelia Di Seclì, Ilaria Boselli, Tiziana Cadossi - Langhirano (PR); Albino Massidda, Gisella Meloni, Claudia Putzu, Carlo Cozzi - Lanusei (NU); Raffaella Buzzetti, Camillo Gnessi, Gaetano Leto, Chiara Foffi, Laura Cipolloni, Chiara Venditti, Chiara Moretti, Angela Carlone - Latina; Aldo Giuseppe Morea, Lucia Bondesan, Sandro Perbellini - Legnago (VR); Aurora Valicenti, Stefania Bertoli, Sabrina Cosimi, Sandra Bianchini - Lido di Camaiore (LU); Stefano Genovese, Valeria Valdes, Dina Miele - Limbiate (MB); Graziano Di Cianni, Paola Orsini, Anna Turco, Cristina Lencioni, Chiara Goretti, Claudia Sannino, Paolo Lemmi - Livorno; Maura Rosco - Locorotondo (BA); Alberto di Carlo, Ilaria Casadidio, Ilaria Cuccuru, Laura Maria Chiara Giorgi, Alba Galli, Marzia Piacentini, Elisa Del Bianco, Chiara Russo, Piera Baroni, Antonella Salvini, Vito Michele Cassano - Lucca; Piero Melandri, Paolo Di Bartolo - Lugo (RA); Gabriele Maolo, Barbara Polenta, Nadia Piccinini - Macerata; Maria Maddalena Atzeni, Cristina Porru - Macomer (NU); Cesare Vincenti, Paola Mega, Enza Magurano, Nicola Pastore, Antonella Cananiello - Maglie (LE); Giuliano Gaspardo, Elettra Brussa Toi, Ciro Antonio Francescutto, Luisa Angeli, Lorena Ronchese - Maniago (PN); Stefano Fazion, Francesca Saggiani, Linneo Enzo Mantovani, Adriana Forapani, Raffaella Antoniazzi, Paola Cristianini, Rita Mazzali, Elena Pierobon - Mantova; Salvatore Turco, Anna Amelia Turco, Maria Teresa Fernicola, Giuseppe Cudemo, Giovanna Taglialatela - Marano di Napoli (NA); Luigi Sciangula, Alessandra Ciucci, Myriam Gandolfo, Baldassarre Grassa, Adele Tono, Rosalia De Marco, Emanuela Simona Olivo, Elisa Bellini - Mariano Comense (CO); Antonino Lo Presti, Antonietta Maria Scarpitta - Marsala (TP); Maria Adelaide Dolci, Fabio Baccetti, Giovanna Gregori, Mary Mori - Massa (MS); Alessandro de Palma, Massimo Alessandri, Paola Sarenari, Cecilia Panni, Ornella Vagnetti, Carla Petri - Massa Marittima (Gr); Angelo Venezia, Roberto Morea, Giuseppe Lagonigro, Giovanni Copeta, Valeria Iannucci, Vittoria Milano, Maria Trupo - Matera; Alberto Casartelli, Giampaolo Scollo - Menaggio (CO); Paolo Emilio Marchetto, Andreas Lochmann, Gianpiero Incelli, Simon Rauch, Anita Stadler, Sonja Breitenberger, Maria Magdalena Steiger, Maria Anna Gamper, Manuela Holzner - Merano (BZ); Claudio, Lambiase, Teresa Di Vece , Maurizio D'Aniello, Luigi Gargiulo, Anna Vitale, Massimo Fezza, Carmela Giordano, Flora Leo - Mercato S. Severino (SA); Antonino Di Benedetto, Domenico Cucinotta, Giacoma Di Vieste, Basilio Pintaudi, Giuseppina Russo - Messina; Giovanni Saitta - Messina; Manuela Moise - Mestre (VE); Lucilla D Monti, Emanuele Bosi - Milano; Nicoletta Musacchio, Annalisa Giancaterini, Augusto Lovagnini Scher, Ilaria Ciullo, Laura Pessina, Silvia Maino, Rosanna Gaiofatto - Milano; Giampaolo Testori, Pietro A Rampini, Nadia Cerutti, Paola S Morpurgo, Giacomo Bonino, Francesca Morreale - Milano; Alfredo Zocca, Barbara Aiello, Maurizio Picca - Milano; Loris Bortolato, Alessandra Cosma, Giuseppe Donà - Mirano (VE); Giuseppe Campobasso - Modugno (BA); Francesco Mario Gentile, Giovanna Mazzotta - Mola di Bari (BA); Marco Comoglio, Roberta Manti, Carlo Bruno Giorda - Moncalieri (TO); Carla Tortul, Roberto Da Ros, Raimonda Muraro, Silvana Carlucci, Lorena Narduzzi, Laura Stanic, Elisabetta Bain, Anna Martignon, Monique Pellegrin - Monfalcone (GO); Giuseppe Panebianco, Federica Tadiotto, Virgilio Da Tos, Michele D'Ambrosio, Monica Faoncella, Marj Zogno, Cristina Lunardi, Patrizia Crivellaro, Loretta Trivellato - Monselice (PD); Giuseppe Panebianco, Federica Tadiotto, Virgilio Da Tos, Michele D'Ambrosio, Monica Faoncella, Marj Zogno, cristina lunardi, Patrizia crivellaro, Loretta Trivellato - Montagnana (PD); Antonio Volpi, Anna Coracina, Anna Maria Cospite - Montebelluna (TV); Valeria Manicardi, Massimo Michelini, Lorenzo Finardi, Francesca Borghi, Elisa Manicardi, Rosa Trianni - Montecchio Emilia (RE); Simonetta Lombardi, Michele Iaccarino, Silvana Costa, Chiara Tommasi, Sabrina Cozza, Federica Marini, Isabella Mecenero, Stefania Massignani - Montecchio Maggiore (VI); Marco Giorgio Baroni, Efisio Cossu, Elena Loy, Marta Tuveri - Moserrato (CA); Paola Pisanu - Muravera (ca); Vincenzo Armentano, Oreste Egione, Sergio Galdieri, Anna Velotti, Gemma Annicelli, Francesco De Lillo, Antonino Azzolina - Napoli; Giuseppe Cozzolino - Napoli; Angelo Foglia, Giovanna Monfregola - Napoli; Adriano Gatti, Raffaele Giannettino, Michele Bonavita, Eugenio Creso - Napoli; Sandro Gentile, Giuseppina Guarino, Giampiero Marino, Adele Esposito - Napoli; Nicolangelo Iazzetta, Adriana Cammarota - Napoli; Nicolangelo Iazzetta, Claudio Giannattasio - Napoli; Salvatore Turco, Ciro Iovine, Anna Amelia Turco, Gabriele Riccardi - Napoli; Luciano Zenari, Lorenzo Bertolini, Claudia Sorgato, Francesca Grippaldi - Negrar (VR); Mauro Stroppiana, Silvia Abate, Natalia Carbone, Iolanda Feccia, Gian Carla Gaggero, Rosa Popolizio, Roberta Severino - Nizza Monferrato (AT); Marco Strazzabosco, Elisabetta Brun, Maria Simoncini, Chiara Alberta Mesturino, Francesco Zen - Noventa Vicentina (VI); Giovanni Paolo Carlesi, Simona Garrone - Novi Ligure (AL); Alfonso Gigante, Anna Maria Cicalò, Rossella Cau, Concetta Clausi - Nuoro; Agostino Paccagnella, Maria Sambataro, Edward Kiwanuka, Tiziana Citro, Barbara Almoto, Eros Bagolin, Daniela Pizzolato, Alessandra Mauri - Oderzo (TV); Alberto Manconi, Giancarlo Tonolo, Giovanni Domenico, Filigheddu, Angela Assunta Pinna, Antonello Carboni, Maria Filippina Angius, Lucia Canu, Sara Cherchi - Olbia (OT); Silvia Calebich, Cinzia Burlotti, Alfonso Piccoli - Ome (BS); Giuseppe Saglietti - Omegna (VB); Sergio Cabras, Barbara Figus, Ignazia Oppo - Oristano; Francesco Mastinu, Maria Franca Mulas, Gianfranco Madau, Marina Cossu, Simonetta Zoccheddu, Marilena Atzeni, Gabriele Idda, Monica Obinu - Oristano; Giovanni Cicioni, Chiara Di Loreto, Massimo Bracaccia - Orvieto (TR); Mario Balsanelli, Mauro Fetonti, Paola Sambo - Ostia (RM); Tommasina Sorrentino, Andrea Del Buono, Iole Gaeta, Angelo Annunziata, Pietro Cannavale, Nunziata Di Palma, Carolina Federico - Ottaviano (NA); Elio Secchi, Maria Antonietta Angotzi, Salvatore Loddoni, Irene Brundu, Franca Careddu, Antonietta Becciu, Gabriella Piras - Ozieri (SS); Giuseppe Torchio, Adolfo Carlo Alberto Bianchi, Gian Battista Colucci, Patrizia Palumbo, Barbara Saviori - Paderno Dugnano (MI); Angelo Avogaro, Daniela Bruttomesso, Cristina Crepaldi, Giampaolo Fadini, Gabriella Guarnieri, Saula de Kreutzenberg, Alberto Maran, Monica Vedovato - Padova; Annunziata Lapolla, Rosanna Toniato, Giuseppe Bax, Barbara Bonsembiante, Claudio Cardone, Maria Grazia Dalfrà, Alessandra Gallo, Michela Masin, Francesco Piarulli, Antonino Pipitone, Giovanni Sartore - Padova; Francesco D'Agati, Lorenza Patrizia Cipolla, Antonella Biondo - Palermo; Maria Antonella Fulantelli - Palermo; Daniela Gioia, Michela Conti - Palermo; Giuseppe Mattina, Lorenza Patrizia Cipolla - Palermo; Giuseppe Mattina, Maria Vaccaro, Iana Costa - Palermo; Giovanni Ridola - Palermo; Giovanni Grossi, Fiorella De Berardinis, Rosanna Piro, Anna Semaforico, Assunta Montalto - Paola (CS); Maria Cristina Cimicchi, Diletta Ugolotti, Daina Filippi, Marina Ferrari - Parma; Sergio Michele Tardio, Maria Cristina Calderini, Maria Grazia Magotti, Laura Franzini, Raffaele Napolitano - Parma; Ivana Zavaroni, Alessandra Dei Cas, Elisa Usberti, Valentina Ridolfi, Monica Antonini, Michela Marina, Elisabetta Marchesi, Nadia Anelli - Parma; Paola Del Sindaco, Enio Picchio, Gianluigi Guercini, Laura Piastrella, Massimo Arcelli - Perugia; Adriano Spalluto, Luigi Maggiulli, Massimo Badiali - Pesaro; Giuliana La Penna, Agostino Consoli - Pescara; Roberto Anichini, Lucia Loredana Merluzzo, Elena Ceccanti, Alessia Lazzarini, Milva Lazzeretti, Irene Howard, Secondina Viti, Alice Magiar - Pescia (PT); Anna Rosa Bogazzi, Giovanna Bendinelli, Massimo Rivelli, Cristina Vietti - Pianezza (TO); Lucia Briatore, Giacomo Calvo, Brunella Falco - Pietra Ligure (SV); Carlo Antona, Daniele De Vido - Pieve di Cadore (BL); Alessandro Ozzello, Enrico Pergolizzi, Daniela Gaia, Paola Gennari, Giuliana Micali - Pinerolo (TO); Valerio Gherardini, Leonardo Moretti, Francesca Iovine, Chiara Goretti, Monica Bientinesi, Luciana Landi - Piombino (LI); Roberto Miccoli, Cristina Bianchi, Giuseppe Penno, Francesca Venditti, Stefano Del Prato - Pisa; Roberto Anichini, Alessandra De Bellis, Anna Tedeschi, Lisetta Butelli, Rossella Picciafuochi, Manola Gioffredi, Roberto Gori, Tiziana Bruschi, Raffaella Malagoli - Pistoia; Roberto Giubbolini, Sfefania Machetti, Susanna Sansi Roasamaria Grisanti - Poggibonsi (SI); Emilia Martedì, Francesco Nappo - Portici (NA); Gaetana Cossu, Pietro Deliperi - Porto Torres (SS); Cristiano Fongher, Anna Gnan - Porto Viro (RO); Giovanna Villani, Claudia Cancro, Stefano Anelli - Portoferraio (LI); Massimo Moretti, Milena Zanon, Anna Battiston, Anna Del Bianco, Michela Signorato - Portogruaro (RO); Vincenzo Mazzini, Marina Orlandi, Maria Cristina Pollace - Portomaggiore (FE); Armando Zampino, Rosa Sinisi, Maria Natale, Giuseppe Citro - Potenza; Maria Calabrese, Lucia Ianni, Monica Lorenzetti, Angela Marsocci, Sandra Guizzotti, Ida Innocenti, Annalia Noci, Ahoua Sagnon, Romina Fioravanti, Paola Mestrini - Prato; Giosuè Ghilardi, Patrizia Fiorina - Priario (BG); Salvatore Turco, Antongiulio Faggiano, Giovanna Donnarumma, Nicola Tafuri, Mariella Andretti, Barbara Tramontano - Quarto (NA); Francesco Cabasino, Fernando Farci, Alberto Atzori, Mariangela Ghiani, Irene Siotto, Marianna Sedda, Alì Manis, Carmela Loddo, Ilaria Loddo, Lucia Pisano, Paola Seguro, Annamaria Cuomo, Lucilla Orlando, Giovanni Battista Olanda - Quartu S'Elena (CA); Michelina Massenzo - Quattromiglia di Rende (CS); Achiropita Pucci, Barbara Greco - Quattromiglia di Rende (CS); Paolo Di Bartolo, Cipriana Sardu - Ravenna; Giovanni Careddu, Raffaella Costa, Vilma Campora, Laura Anselmi, Andrea Corsi - Recco (GE); Celestino Giovannini - Reggio Calabria ; Domenico Mannino, Emira Dal Moro, Maurizio Postorino, Eugenio Alessi - Reggio Calabria ; Giovanni Perrone, Francesca Corazziere, Irene La Puzza - Reggio Calabria ; Pier Francesco Tripodi, Angela Mirto, Sergio Grasso, Giulia Zerbi-Cama, Antonella Giampaolo - Reggio Calabria ; Valeria Manicardi, Prisco Sborbone, Eliana Gardini, Elisa Manicardi, Clelia Di Seclì, Patrizia Marrino, Rosa Trianni, Marina Greci - Reggio Emilia; Emilio Rastelli, Claudia Rastelli - Riccione (RN); Basilio Battisti, Anna Rita Aleandri, Maria Virginia Guidi, Rosaria Faraglia, Verena Lilli - Rieti; Rocco Bulzomì - Roma; Francesco Chiaramonte, Renato Giordano, Maria Giuseppina Migneco, Mauro Rossini, Natalia Visalli, Daniela Danna, Silvana Baldassarra, Romina Ralli, Samanta Roma, Orietta Pannozzo, Alessandra Cignetti, Bruna Facchiani, Claudia Righini - Roma; Mariano Pio D'Accinni, Donatella Paolucci, Anna D'Ubaldi, Maria Teresa D'Angelo, Manuela Fratini, Giovanni Masaro, Marco Pietrantoni, Roberta La Rosa - Roma; Maria Giuliano, Maria Grazia Pennafina, Pasquale Di Perna - Roma; Sergio Leotta, Concetta Suraci, Natalia Visalli, Roberto Gagliardi, Lucia Fontana, Maria Altomare, Silvia Carletti, Santina Abbruzzese, Letizia Bruschi, Roberta Lancione - Roma; Sebastiano Filetti, Susanna Morano, Marcella Santilli, Elisabetta Mandosi, Mara Fallarino, Marco Rossetti - Roma; Fabio Piergiovanni, Fiorella Massimiani, Angela Simonetta, Danila Fava, Fulvia De Luca, Giuliana Leacche, Teresa Giampietro, Rossella Guarino - Roma; Maurizio Poggi, Anna Penza, Francesca Piccirilli, Roberta Pisano, Annamaria Prioletta, Caterina Saponara - Roma; Raffaele Scalpone, Fabrizia Toscanella, Alessandro Scoppola, Lorena Mancini - Roma; Claudio Tubili, Lelio Morviducci, Maria Rosaria Nardone, Paola Fratoni - Roma; Daniela Cappelloni, Lina Lardieri, Alessandro Urbani, Tiziana Santucci, Gabriella del Monte, Irene Pellegrino, Franca Rauseo, Patrizia Alini - Roma; Giuseppe Armentano, Maria Grazia Restuccia, Giuseppe Vatrano - Rossano (CS); Renzo Girardello, Renzo Gennaro, Lorena De Moliner, Annalisa Mattuzzi, Katja Speese,Elena Bettini, Fabiola Frisinghelli, Monica Falqui - Rovereto (TN); Giacomo Vespasiani, Illidio Meloncelli, Lina Clementi, Marianna Galetta, Valentina Marconi - S. Benedetto del Tronto (AP); Paolo Bordin, Maurizio Sacandi, Laura Perale, Luciana Dotto, Roberto Da Ros, Nella Pupin, Elena Rosso, Fabiola Zamparo - S. Daniele del Friuli (UD); Silvestre Cervone, Matteo Pontonio, Lucia De Angelis - S. Marco in Lamis (FG); Manola Nicoletti, Nazzareno Trojan, Tiziano Croatto, Rita Centis - S. Vito al Tagliamento (PN); Patrizia Li Volsi, Elisa Levis, Giorgio Zanette - Sacile (PN); Carmela Vinci, Milena Zanon, Loredana Geretto, Cristina Toffolo, Maria Grazia Furlan - San Donà di Piave (VE); Vincenzo Sica, Raffaella Derai, Marina Armeni, Anna Bruna Urraci, Letizia Carreras, Elisabetta Cossu, Simonetta Mamusa, Maria Antonietta Pisano - San Gavino Monreale (VS); Salvatore De Cosmo, Anna Rauseo - San Giovanni Rotondo (FG); Marco Strazzabosco, Elisabetta Brun, Maria Simoncini, Chiara Alberta Mesturino, Francesco Zen - Sandrigo (VI); Roberto Sturaro, Maurizio Raffa, Federico Quattrocchi - Sanremo (IM); Anna Carbone, Cristina Gnocchi, Maria Rosa Anelli, Ester Fontana, Giuliana Alfoli - Sant' Angelo Lodigiano (LO); Maura Rosco, Giovanni Labarile - Santeramo (BA); Fabrizia Caucci - Sassari; Luca Lione, Dino Dessì - Savona; Mario Monachesi, Giovanni Carta, Mara Boschetti, Enrica Ceresola, Emanuela Venier - Savona; Stefano De Riu, Umberto Amelia - Scafati (SA); Francesco Calcaterra, Fedele Cataldi, Marina Miola - Schio (VI); Silvana Manfrini, Silvia Rilli, Gessica Tinti - Senigallia (AN); Alessio Lai, Barbara Locci, Donatella Putzu - Senorbi (CA); Stefano Genovese, Loredana Bucciarelli, Maurizio Rondinelli, Monica Bulgheroni - Sesto S. Giovanni (MI); Stefano Gonnelli, Alice Cadirni, Italo Tanganelli - Siena; Karl Egger, Kerstin hofmann, Margareth Tanner, Heidrun Hohenegger - Silandro (BZ); Cristina Porru, Angela Canneddu, Mercedes Porcu - Siniscola (NU); Vittoria Sesta, Franca Daidone - Siracusa; Luigi Vincis, Viviana Orlandi, Cinzia Pilloni, Rossana Farci, Ilaria Pelligra, Giuseppina Renier - Sirai - Carbonia (CI); Marco Mameli, Elvira Devigus, Anna Pala - Sorgono (NU); Giuseppe Felace, Ida Fumagalli, Paola Tosoni, Daniela Ovan, Silvia Giacomello, Nada Peressini, Fiorella Rossi - Spilimbergo (PN); Carlo Lalli, Mafalda Agliani, Maria Luisa Picchio, Maura Scarponi, Ludovico De Pascalis, Erika Bonucci - Spoleto (PG); Francesco Malci, Anita De Ciocchis, Chiara Moscatelli - Subiaco (RM); Barbara Macerola, Angelina Mandarino, Sandra Incani - Sulmona (AQ); Silvano Davì, Maria Ausilia Caccavale, Simona Borla, Marina Pognant Gros - Susa (TO); Ennio Lattanzi, Concettina Piersanti, Anna Piersanti, Irene Spinelli, Gabriella Quaranta, Silvia D'Ascanio - Teramo; Lorenzo De Candia - Terlizzi (BA); Antimo Aiello, Pietro Di Caro, Fabrizia Flocco, Agnese Franco, Maria Antonietta Lezzi, Anna Meo, Antonella Meo, Alfredo Puntillo, Giovanna Scarpina, Marco Tagliaferri, Celeste Vitale - Termoli (CB); Giovanni Cicioni, Maria Grazia Massarelli, Stefania Venturi - Terni; Augusto Travaglini, Patrizia Draghi - Terni; Elisa Forte, Lucilla Tamburro, Catia Palmacci, Maria Palma Abbate - Terracina (LT); Stefania Deiana, Francesca Scanu - Thiesi (SS); Paolo Pomante - Tocco da Casauria (PE); Alberto Bruno, Paolo Cavallo Perin, Ezio Ghigo, Massimo Porta, Paola Scuntero, Rosanna Arcari, Silvana Bertaina, Simona Bo, Fabio Broglio, Graziella Bruno, Mariella Degiovanni, Paolo Fornengo, Giorgio Grassi, Valeria Inglese, Mauro Maccario, Giorgio Maghenzani, Saverio Marena, Valentino Martina, Pietro Passera, Gianluca Ruiu, Milena Tagliabue, Maria Zanone - Torino; Aurora Grassi, Alberto Mormile, Anna Maria Ingaramo, Pietro Demurtas - Torino; Luca Monge, Gian Mario Boffano, Katia Macrì, Paola Maio - Torino; Luca Richiardi, Alessandra Clerico - Torino; Cesare Dossena, Simona Bosoni - Tortona (AL); Francesca Novara, Daniela Mancuso, Francesca Cipro - Trapani; Paolo Acler, Tiziana Romanelli, Sandro Inchiostro, Massimo Orrasch, Cristina Faes, Silvia Clementi, Franca Endrizzi, Ilaria Nicolao - Trento; Carlo Antonio Bossi, Giancarla Meregalli, Annalisa Balini, Denise Berzi, Anna Pulcina - Treviglio (BG); Agostino Paccagnella, Maria Sambataro, Edward Kiwanuka, Tiziana Citro, Barbara Almoto, Eros Bagolin, Daniela Pizzolato, Alessandra Mauri - Treviso; Riccardo Candido, Elena Manca, Alessandra Petrucco, Elisabetta Caroli, Elisabetta Tommasi, Giuseppe Jagodnik, Nevia Daris, Rossella Alberti, Katja Tercelj, Elisabetta Presti, Sarah Perini, Elisa Del Forno - Trieste; Maria Antonietta Pellegrini, Laura Tonutti, Giorgio Venturini,Sandra Agus, Franco Grimaldi - Udine; Mauro Andreani, Federica Turchi, Maurizio Sudano, Gigliola Martinelli, Stefania Lani - Urbino (PU); Francesca Innelli, Piera Grammaldo, Angelo Vistocco - Vallo della Lucania (SA); Silvio Sposito, Renzo Rongioletti, Maria Candidi - Velletri (RM); Anna Rosa Bogazzi, Giovanna Bendinelli, Massimo Rivelli, Cristina Vietti - Venaria Reale (TO); Ermanno Moro, Antonella Senesi - Venezia; Giuseppe Saglietti, Giuseppe Placentino, Antonella Schellino - Verbania Pallanza (VB); Roberto Mingardi, Luciano Lora, Cristina Stocchiero, Rachele Reitano, Mee Jung Mattarello, Giovanni Ronzani, Silvia Pegoraro, Matteo Rolandi, Veronica Baggio, Elisa Rancan - Vicenza; Marco Strazzabosco, Elisabetta Brun, Maria Simoncini, Chiara Alberta Mesturino, Francesco Zen - Vicenza; Mauro Giovanni Schiesaro, Giovanni Vita, Beatrice Cenci, Vania ampostrini, Ilaria Basso, Augusta Turrini - Villafranca Veronese (VR); Paola Pisanu - Villasimius (CA); Ida Mangone, Enrico Cazzaniga, Giovanni Sifarelli - Vimercate (MB); Claudio Grande, Claudia Arnaldi, Paolo Fiorentini, Enrico Capotorti, Angela Del Prete - Viterbo; Umberto Iannaccone, Mauro Ragonese, Caterina Scoponi, Laura Tilaro, Silvia Pelliccioni, Rossana Slongo, Emanuela Vita - Viterbo; Arcangela Garofalo, Filippo Vitale, Giuseppina Saggio, Biagia Campanella - Vittoria (RG); Andrea Del Buono, Tommasina Sorrentino, Teresa Borrelli, Mariarosaria Matrecano - Volla (NA); Aldo Giuseppe Morea, Lucia Bondesan, Sandro Perbellini - Zevio (VR).
