# Supplementary figures and images for: Gender-Disparities in Adults with Type 1 Diabetes: More Than a Quality of Care Issue. A Cross-Sectional Observational Study from the AMD Annals Initiative
Source: PLoS One. 2016 Oct 3;11(10):e0162960. doi: 10.1371/journal.pone.0162960 (PMC5047461; doi:10.1371/journal.pone.0162960)

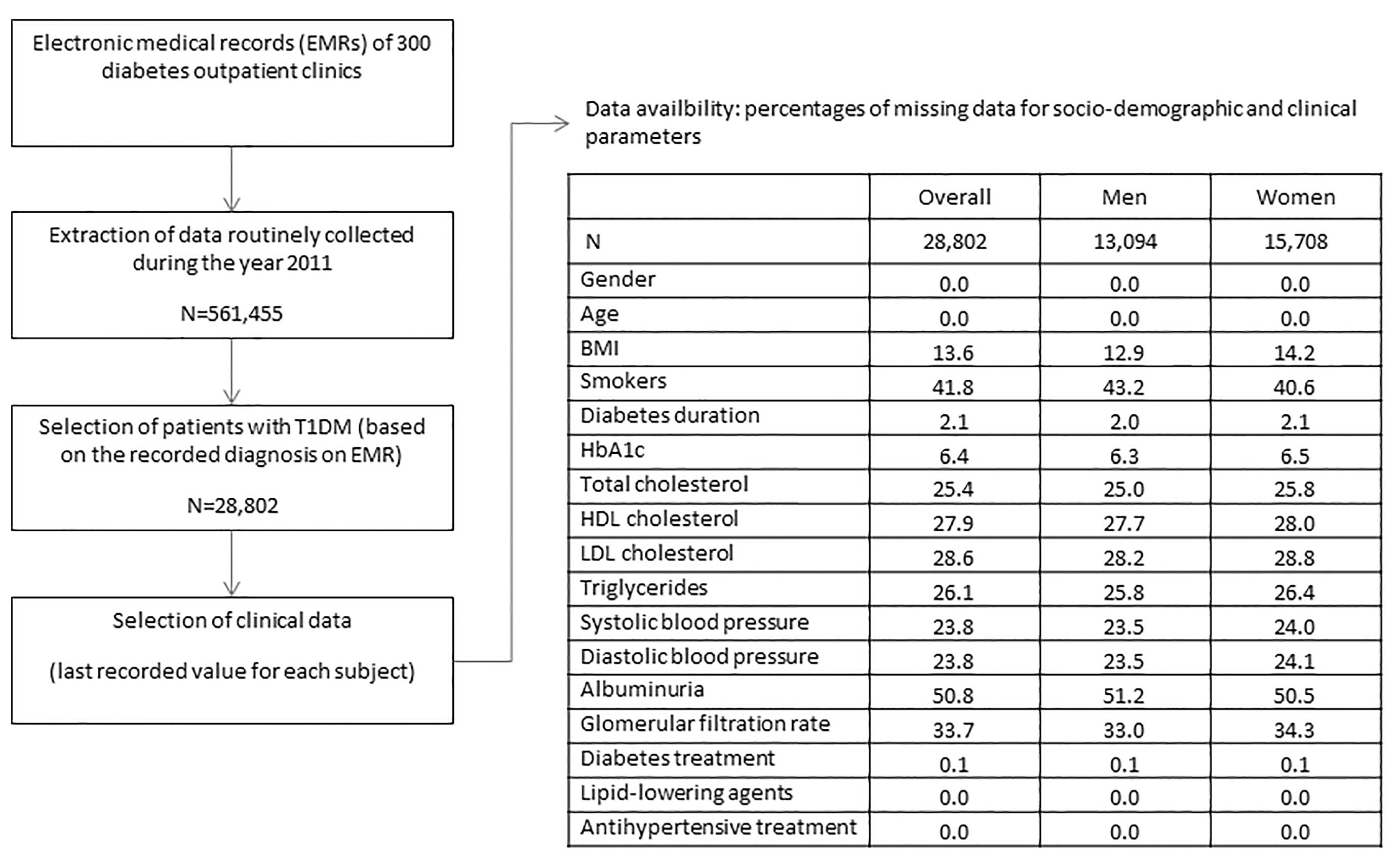

Supplement: S1 Fig — (TIF) [file pone.0162960.s002.tif]
